# Concanavalin A/IFN-Gamma Triggers Autophagy-Related Necrotic Hepatocyte Death through IRGM1-Mediated Lysosomal Membrane Disruption

**DOI:** 10.1371/journal.pone.0028323

**Published:** 2011-12-05

**Authors:** Chih-Peng Chang, Ming-Chen Yang, Huan-Yao Lei

**Affiliations:** 1 Department of Microbiology and Immunology, College of Medicine, National Cheng Kung University, Tainan, Taiwan; 2 Institute of Basic Medical Sciences, College of Medicine, National Cheng Kung University, Tainan, Taiwan; 3 Infectious Disease and Signaling Research Center, College of Medicine, National Cheng Kung University, Tainan, Taiwan; Universidade Federal do Rio de Janeiro, Brazil

## Abstract

Interferon-gamma (IFN-γ), a potent Th1 cytokine with multiple biological functions, can induce autophagy to enhance the clearance of the invading microorganism or cause cell death. We have reported that Concanavalin A (Con A) can cause autophagic cell death in hepatocytes and induce both T cell-dependent and -independent acute hepatitis in immunocompetent and immunodeficient mice, respectively. Although IFN-γ is known to enhance liver injury in Con A-induced hepatitis, its role in autophagy-related hepatocyte death is not clear. In this study we report that IFN-γ can enhance Con A-induced autophagic flux and cell death in hepatoma cell lines. A necrotic cell death with increased lysosomal membrane permeabilization (LMP) is observed in Con A-treated hepatoma cells in the presence of IFN-γ. Cathepsin B and L were released from lysosomes to cause cell death. Furthermore, IFN-γ induces immunity related GTPase family M member 1(IRGM1) translocation to lysosomes and prolongs its activity in Con A-treated hepatoma cells. Knockdown of IRGM1 inhibits the IFN-γ/Con A-induced LMP change and cell death. Furthermore, IFN-γ^−/−^ mice are resistant to Con A-induced autophagy-associated necrotic hepatocyte death. We conclude that IFN-γ enhances Con A-induced autophagic flux and causes an IRGM1-dependent lysosome-mediated necrotic cell death in hepatocytes.

## Introduction

Programmed cell death has been classified as type I apoptosis and type II autophagy-associated cell death [1]. Autophagy is an evolutionarily conserved lysosomal pathway to generate energy by digesting cytoplasmic proteins and organelles. Although autophagy allows cells to survive from starvation or stress, an extended or massive autophagy process turns to kill cells [2], [3], [4]. Necrosis is usually considered as a non-programmed cell death without features of apoptosis and autophagy [5], [6]. However, necrosis can also be regulated and controlled by cellular proteins, such as lysosomal protease cathepsins [7]. Programmed cell death can switch to necrosis by inhibition of specific proteins of apoptosis or autophagy [6]. Cross-regulation between these different types of cell death remains unclear.

Lysosomes are intracellular organelles which contain various hydrolytic enzymes to control turnover of cellular macromolecules and eliminate intracellular pathogens [8]. However, they can also be responsible for mediating programmed cell death or necrosis [9]. Lysosome membrane permeablization (LMP) changes will initiate a lysosome-mediated cell death. When the lysosome membrane is damaged through LMP, cathepsins or other hydrolytic enzymes will be released from lysosomes to the cytosol and cause cell death [10]. Autophagy is a lysosome-dependent pathway and can cause a type II programmed cell death. Accumulated lysosomes or autophagolysosomes are increased in autophagy-associated cell death. However, the role of LMP on autophagy-associated cell death is poorly understood.

Interferon-gamma (IFN-γ), a major Th1 cytokine, is an important mediator for immune-mediated hepatitis [11], [12]. A p53-dependent cell cycle arrest and apoptosis caused by IFN-γ in hepatocytes is suggested [13]. However, IFN-γ can also regulate the autophagic response to eliminate intracellular pathogen infection in macrophages or cause cell death of epithelial cells [14], [15]. The immunity related GTPase family proteins (IRG), particularly IRGM1, have been considered as key mediators for IFN-γ-induced autophagy [16]. We have reported that Concanavalin A (Con A) can induce both T cell-dependent and T cell-independent acute hepatitis [17]. A Bcl-2/adenovirus E1B 19 kDa-interacting protein 3 (BNIP3)-related mitochondria autophagy was involved in the hepatocyte death [18]. In this study, we further report that IFN-γ can enhance Con A-induced autophagic flux and hepatocyte death. The enhanced cell death by IFN-γ/Con A represents a lysosomal proteases, cathepsin B- and cathepsin L-dependent necrosis. IRGM1 participates in this IFN-γ-stimulated LMP-associated necrosis in Con A-treated hepatocytes.

## Results

### IFN-γ enhances autophagic flux and causes necrotic type cell death in Con A-treated hepatocytes

**Figure 1:**
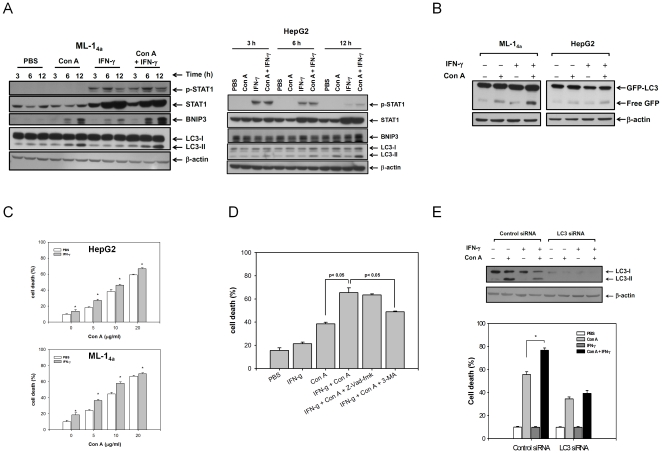
IFN-γ enhances autophagic flux and cell death in Con A -treated hepatoma cell lines. **A**. Con A/IFN-γ induces autophagy in hepatoma cells. ML-1_4a_ and HepG2 cells were treated with PBS, Con A, IFN-γ or IFN-γ/Con A and then collected cell lysates at indicated time. The expressions of phospho-STAT1, STAT1, BNIP3, LC3-I and LC3-II were determined by Western blot. **B**. Generation of free GFP from pGFP-LC3-expressed hepatoma cells by ConA/IFN-γ treatment. pGFP-LC3-transfected ML-1_4a_ or HepG2 cells were treated with Con A and IFN-γ for 24 hours. The cell lysates were extracted to determine expression of GFP-LC3 and free GFP by Western blot. **C**, IFN-γ enhances Con A-induced cell death in hepatoma cells. HepG2 and ML-1_4a_ cells were treated with various concentrations of Con A in the presence or absence of IFN-γ for 24 hours. Cell death was determined by propidium iodide staining and analyzed by flow cytometry. **D and E**. IFN-γ enhances autophagy-dependent cell death in Con A-treated hepatoma cells. ML-1_4a_ cells were pre-incubated with 3-methyladenine or Z-Vad-fmk (**D**), or transfected with control or LC3 siRNA (**E**). Then these cells were co-treated with Con A and IFN-γ for 24 hours. The knock down effect of LC3 was monitored by Western blot. The cell death of all treatments was determined by propidium iodide staining. All results are representative of two to three experiments. * shows statistically significant differences relative to PBS group. (p<0.05).

**Figure 2:**
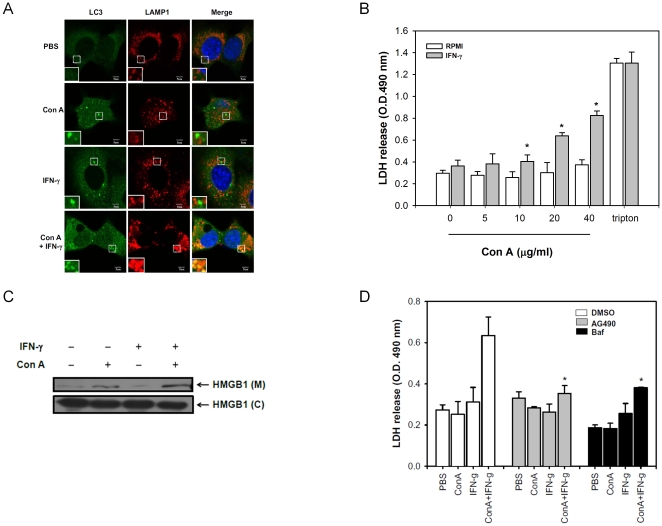
IFN-γ causes necrotic type cell death in Con A-treated hepatoma cell lines. **A**. The colocalization of punctate LC3 and LAMP-1 in ML-1_4a_ cells. ML-1_4a_ cells were treated with PBS, Con A, IFN-γ or IFN-γ/Con A for 6 hours. All cells were fixed and stained with anti-LC3 or LAMP1 antibodies. The colocalization of punctate LC3 and LAMP 1 was assayed using confocal microscopy. Cell nucleus was stained with Hoechst (blue). **B and C**. IFN-γ triggers LDH and HMGB1 release from Con A-treated hepatoma cells. ML-1_4a_ was treated with Con A at various doses in the presence or absence of IFN-γ for 24 hours. The cell supernatants were then collected, the LDH (**B**) was determined with enzymatic activity as described in Materials and Methods while the HMGB1 (**C**) was determined by Western blot. C: total cell lysate; M: culture supernatant in media. **D**. IFN-γ-caused LDH release in Con A-treated hepatoma cells is JAK2- and autophagy-dependent. ML-1_4a_ cells were pre-incubated with DMSO, AG490 or bafilomycin A1 (baf) for 1 hour and then co-treated with Con A and IFN-γ for another 24 hours. The activity of LDH in cell culture supernatants was determined. All results are representative of two to three experiments.* shows statistically significant differences relative to RPMI or DMSO group. (p<0.05).

Autophagic flux is a continuing process from autophagosome generation to cargo degradation. We have reported that Con A induced an acute hepatitis via a BNIP3 dependent mitochondria autophagic pathway in hepatocytes [18]. The double membrane autophagosomes can be found in Con A-treated ML-1_4a_ cells but reduced in the presence of autophagy inhibitor, 3-methyladenine, from electron microscopy (Figure S1). This pathway is further extended to autophagic flux and is enhanced by IFN-γ. The BNIP3 was induced by Con A or IFN-γ alone, and enhanced while co-treatment in ML-1_4a_ cells. However, only slightly increase of BNIP3 expression was detected at 12 hours post treatment of IFN-γ/Con A in HepG2 cells, which might be due to the high level of basal BNIP3 expression in HepG2 cells. The LC3-II conversions were both slightly increased in IFN-γ/Con A-treated ML-1_4a_ or HepG2 cells (Figure 1A). We next used GFP-LC3 processing to follow autophagic flux, and an increase of free GFP from GFP-LC3 processing was found in IFN-γ/Con A-treated ML-1_4a_ or HepG2 cells (Figure 1B). This suggests that IFN-γ enhances the autophagic flux on Con A-treated hepatoma cells. We next determined the effect of IFN-γ on Con A-induced autophagic cell death. Con A dose-dependently induced HepG2 and ML-1_4a_ cell death, the addition of IFN-γ significantly enhanced the Con A-induced cell death, especially at the low dose of 5–10 µg/ml of Con A (Figure 1C). The increase of cell death by IFN-γ on Con A-induced cell death was diminished by 3-methyadenine (autophagic inhibitor) or LC3 siRNA, but not by Z-Vad-fmk (apoptosis inhibitor), suggesting that this enhancement in cell death is not due to apoptosis (Figure 1D and 1E). To further confirm this phenomenon, we monitored the fragmented DNA from apoptotic cells by propidium iodide (PI) staining. The apoptotic inducer, puromycin, induced fragmented DNA with increase of sub G1 population in ML-1_4a_ ells. Instead, no such increased-sub G1 population was detected in Con A-, IFN-γ- or IFN-γ/Con A- treated ML-1_4a_ cells, suggesting that these cells were not died by apoptosis (Figure S2). These data indicate that IFN-γ enhances Con A-induced autophagic cell death in hepatocytes. Con A alone-induced cell death can be easily detected by propidium iodide or eosin Y exclusion assay (data not shown), but not by LDH release, a feature of necrotic cell death with detection in the culture supernatant. However, early fusion of autophagosomes and lysosomes by IFN-γ in Con A-treated ML-1_4a_ cells suggested that lysosome-mediated necrosis might be induced (Figure 2A). As results showed in Fig. 2B, we can detect the LDH release in Con A and IFN-γ treated hepatoma cells. Another marker of necrosis, the release of nuclear protein, HMGB1, was also found to be released from Con A -treated hepatoma cells, and IFN-γ could enhanced this release (Figure 2C). Furthermore, IFN-γ can increase Annexin V/PI double positive cell population, which suggests as necrotic cells, of Con A-treated ML-1_4a_ cells, and pretreatment of 3-methyladenin will reduce this enhancement (Figure S3). AG490 (Jak2 inhibitor), or bafilomycin A1 (autophagic flux inhibitor) both inhibited the IFN-γ-triggered LDH release in Con A-treated hepatoma cells (Figure 2D). These results suggest that IFN-γ can enhance Con A-induced autophagic flux and cause a necrotic type cell death.

### IFN-γ causes a necrotic cell death with increase of lysosomal membrane permeabilization (LMP) in Con A-treated hepatocyte

**Figure 3:**
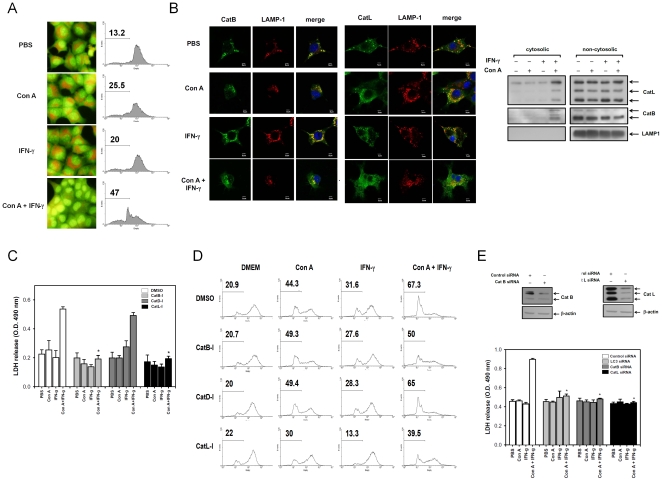
IFN-γ induces a necrotic type cell death with increase of LMP in Con A-treated hepatoma cells. **A**. IFN-γ causes increase of LMP in Con A-treated hepatocytes. ML-1_4a_ cells were stained with acridine orange and treated with Con A and IFN-γ. The red fluoresce of acridine orange was observed in florescence microscope or analyzed by flow cytometry. **B**. IFN-γ induces cathepsin-B/L release in Con A-treated hepatoma cells. After treatment of Con A and IFN-γ for 12 hours, ML-1_4a_ cells were fixed and stained with anti-cathepsin B, anti-cathepsin-L or anti-LAMP1 antibody or extracted the cytosolic proteins by digitonin as described in Materials and Methods. The distribution of cathepsinB/L was analyzed using a confocal fluorescence microscope or immunoblotting. Cell nucleus was stained with Hoechst (blue). **C–E**. Cathepsin B/L mediates IFN-γ-induced LDH release and increase of LMP in Con A-treated hepatoma cells. ML-1_4a_ cells were pretreated with DMSO or different cathepsin inhibitors (**C,D**) or transfected with siRNA of control, LC3, cathepsin B or cathepsin L (**E**), and then treated with Con A and IFN-γ for another 24 hours. The release of LDH and cellular LMP in all treatments were both determined as described above. The knock down effect of cathepsin B and L were monitored by Western blot. All results are representative of two to three experiments.* shows statistically significant differences relative to DMSO group. (p<0.05).

We next determined how IFN-γ enhances Con A-induced necrotic type cell death. The LMP was determined by acridine orange (AO), a lysosomotropic fluorochrome used to measure cellular increase of the LMP. AO-stained normal cells showed red punctate on microscopic observation and intact red fluorescence on flow cytometry. The Con A or IFN-γ treatment alone have the same pattern with that of normal cells. However, IFN-γ plus Con A caused a dramatic loss of the punctate staining and red fluorescence on AO-stained hepatoma cells compared with the Con A or IFN-γ treatment groups (Figure 3A). We also found that the lysosomal proteases, cathepsin B and L, were released into the cytoplasm. They are normally localized in LAMP1-positive lysosomes of ML-1_4a_ cells. After treatment with IFN-γ and Con A, increases of LAMP1-negative cathepsin B or L were found in the cytosol, indicating that cathepsin B and L were released following the IFN-γ plus Con A-induced increment of LMP (Figure 3B). We further determined whether cathepsin can mediate the IFN-γ-triggered necrosis of Con A-treated hepatoma cells. Using several specific inhibitors to block the IFN-γ plus Con A-induced LDH release and LMP increment, we found that inhibitors of cathepsin B (Z-Phe-Phe-CH_2_F) and L (CA-074Me), but not an inhibitor of cathepsin D (pepstatin A), can inhibit the IFN-γ-induced LDH release and increase of LMP in Con A-treated hepatoma cells (Figure 3C and 3D). To further verify the role of cathepsin B, L and autophagy in the IFN-γ-triggered necrotic cell death, the siRNA of cathepsin B, L and LC3 were used. The IFN-γ/Con A induced LDH release was inhibited by cathepsin B, L and LC3 siRNA treatment (Figure 3E). These results suggest that IFN-γ-induced LMP causes cathepsin B and L-dependent necrotic cell type death in Con A-treated hepatoma cells.

### IRGM1 mediates IFN-γ-enhanced LMP-associated lysosomal cell death in Con A-treated hepatocytes

**Figure 4:**
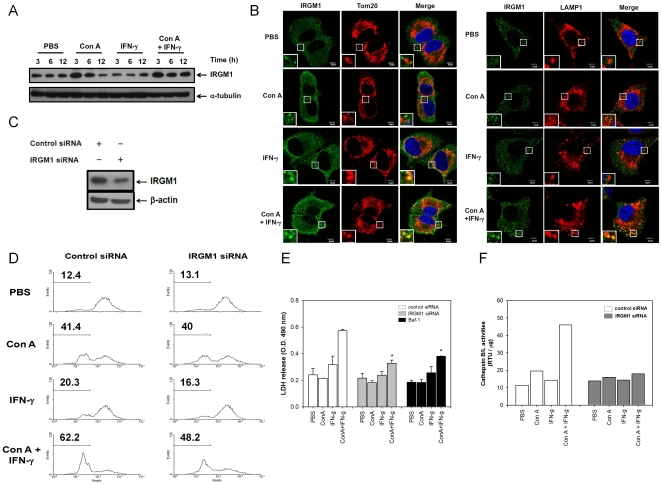
IRGM1 mediates IFN-γ-enhanced LMP-associated necrosis-like cell death in Con A-treated hepatoma cells. **A**. IRGM1 expression in IFN-γ/Con A-treated hepatoma cells. ML-1_4a_ cells were treated with PBS, Con A, IFN-γ or IFN-γ/Con A and collected cell lysates at indicated time. The expression of IRGM1 was determined by Western blot. **B**. IRGM1 translocates to mitochondria and lysosomes. After treatment of Con A or IFN-γ for 12 hours, ML-1_4a_ cells were fixed and stained with anti-IRGM1, Tom20, and LAMP-1 antibodies. The co-localization of IRGM1 and Tom20 or LAMP-1 was analyzed using a confocal fluorescence microscope. Cell nucleus was stained with Hoechst (blue). **C**. Knock down of IRGM1 by siRNA. ML-1_4a_ cells were transduced with siRNA of control or IRGM1 as described in Materials and Methods. The expression of IRGM1 was determined by Western blot. **D**. Knock down of IRGM1 attenuates IFN-γ-enhanced LMP in Con A-treated hepatoma cells. ML-1_4a_ cells were transduced with siRNA of control or IRGM1 and treated with Con A and IFN-γ. The cells were then stained with acridine orange to monitor the LMP. **E**. IRGM1 mediates IFN-γ-induced increase of cytosolic cathepsin B/L activities in Con A-treated hepatoma cells. After knock down of IRGM1 by siRNA, ML-1_4a_ cells were treated with Con A and IFN-γ and the cytosolic fraction was collected. The activities of cytosolic cathepsin B/L were then determined. **F**. IRGM1 mediates IFN-γ-enhanced LDH release from Con A-treated hepatoma cells. ML-1_4a_ cells were pretreated with siRNAs or bafilomycine A1 and then incubated with Con A and IFN-γ. The activity of LDH in cell culture supernatants was determined. All results are representative of two to three experiments.* shows statistically significant differences relative to control group. (p<0.05).

The immunity-related GTPase, IRGM1, is essential for IFN-γ-dependent anti-microbial function and autophagy induction [16]. Since IFN-γ causes an autophagy-associated necrotic cell death in Con A-treated hepatoma cells, we further studied the role of IRGM1 on this phenomenon. To our surprise, Con A, not IFN-γ, enhanced a temporary increase of IRGM1 at 3 hours post treatment in ML1_4a_ cells. However, the increased level of IRGM1 could be sustained to 12 hours in IFN-γ/Con A-treated cells, suggesting the IRGM1 expression were enhanced by IFN-γ (Figure 4A). With confocal microscopy observation, we found that IRGM1 was co-localized with mitochondria-related protein, Tom20, in IFN-γ or IFN-γ/Con A-treated ML-1_4a_ cells, but not in PBS or Con A-treated cells. In addition to mitochondria translocation, IRGM1 was found to co-localize with LAMP1 only in IFN-γ/Con A-treated cells, but not in IFN-γ-treated cells (Figure 4B). These data suggest that IFN-γ/Con A enhance not only the IRGM1 expression, but also translocation of IRGM1 to lysosome. Furthermore, the IRGM1 expression was knocked down by siRNA to further investigate its role in IFN-γ/Con A-induced LMP-associated necrosis (Figure 4C).We found that the IFN-γ/Con A-caused increase of LMP in hepatoma cells was significantly inhibited by IRGM1 siRNA, suggesting that IRGM1 is involved in IFN-γ/Con A-caused LMP change (Figure 4D). Because the activity of cathepsin B and L is crucial for IFN-γ/Con A-caused LMP change and necrotic cell death as shown above, and might be regulated by IRGM1. Indeed, the cytosolic cathepsin B/L activity as well as the LDH release were also abolished by IRGM1 siRNA in IFN-γ/Con A-treated hepatoma cells (Figure 4E and 4F). These results suggest that IRGM1 regulates the LMP change that releases the cathepsin B/L to causes lysosomal cell death in IFN-γ/Con A-treated hepatoma cells.

### IFN-γ^−/−^ mice are resistant to Con A-induced autophagy-associated necrotic type cell death

**Figure 5:**
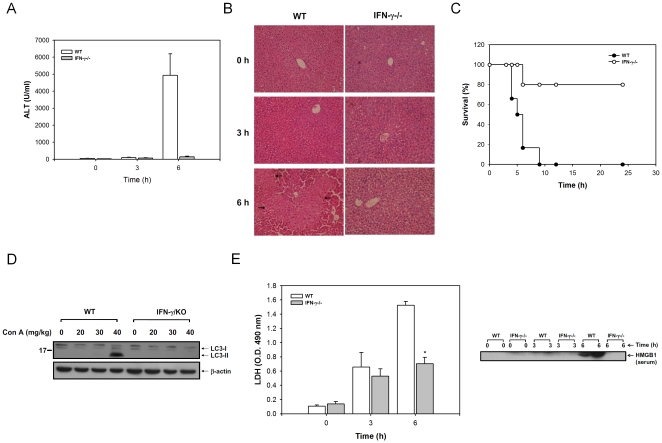
IFN-γ^−/−^ mice are resistant to Con A-induced autophagy-associated necrotic type cell death. **A**. Serum level of ALT on Con A-treated mice. Wide type or IFN-γ^−/−^ mice received intravenous injection of Con A and the serum were collected at indicated time. The serum level of ALT was determined as described in Materials and Methods. (n = 5) **B**. Histological staining of mice liver tissue. The liver tissues of wild type or IFN-γ^−/−^ mice were collected to stain with haematoxylin and eosin at indicated time post Con A injection. The arrow head indicates necrotic hepatocytes. **C**. IFN-γ^−/−^ mice are resistant to Con A-induced mortality. Wild type or IFN-γ^−/−^ mice were injected intravenously with Con A and the survival was monitored. (n = 7) **D**. IFN-γ^−/−^ mice show reduction of Con A-induced autophagy. The liver extracts of wild type or IFN-γ^−/−^ mice were collected to detect the LC3-II conversion by immune blotting at 3 hours post Con A injection. **E**. Serum level of LDH and HMGB1 on Con A-treated mice. Sera of Con A-treated wild type or IFN-γ^−/−^ mice were collected at indicated time. The level of LDH or HMGB1 was determined as described above. (n = 5) All results are representative of two to three experiments. * shows statistically significant differences relative to control group. (p<0.05).

To further evaluate this phenomenon *in vivo*, we compared the Con A-induced hepatitis in IFN-γ^−/−^ mice with that in wild type mice. In wild type mice, an intravenous injection of 40 mg/kg of Con A increased the serum ALT, and necrosis in the liver was observed by histological staining at 6 hours post injection, and the mice finally die within 10 h. In contrast, the IFN-γ^−/−^ mice were resistant to Con A-induced hepatitis, with less mortality, no increase of serum ALT, and no necrosis in the liver (Figure 5A–5C). We also found that the LC3-II conversion was induced in Con A-treated wild type mice, but not in IFN-γ^−/−^ mice (Figure 5D). The serum LDH and HMGB1 were also significantly inhibited in IFN-γ^−/−^ mice comparing with wild type mice at 6 hours post Con A injection (Figure 5E). This suggests that IFN-γ can enhance the Con A-induced necrotic type cell death *in vivo*.

## Discussion

Autophagy is a cell survival system to protect cells from damage by protein aggregates or injured organelles, but it can also lead to cell death with characteristics of apoptosis or necrosis under different conditions. We have previously reported that autophagic induction resulted in hepatocyte death with Con A-induced acute hepatitis. In this study we further showed that IFN-γ, an important mediator for immune-mediated liver injury, enhances the autophagic flux and triggers a lysosomal cell death in Con A-treated hepatocytes. This autophagy-dependent necrotic cell death is mediated by IFN-γ-related IRGM1-induced LMP to release lysosomal proteases, cathepsin B/L.

IFN-γ can induce hepatocytes to undergo cell arrest and apoptosis after in vitro culture for 48 to 72 hours, where an IRF-1-dependent caspase-3 and p53 expression mediates this IFN-γ-caused hepatocyte apoptosis [13]. IFN-γ has also been shown to enhance apoptosis with other cytokines or death-related receptors [19], [20], [21]. In our study of the hepatoma ML-1_4a_ cell line, we found neither any caspase-3 activation, nor any characteristics of apoptosis within 24 hours post IFN-γ treatment. Tagawa Y et al. have reported that IFN-γ would enhance Con A-induced hepatocyte apoptosis on Con A-induced acute hepatitis in mice [22]. In our earlier study, however, we reported that autophagy occurred before the Con A-induced apoptosis in mice [17]. The dose used can explain this discrepancy, as a higher dose (40 mg/kg) of Con A is required to induce autophagy in mice. The LC3-II conversion was found in wild type mice, but not in IFN-γ−/− mice (Figure 5D). The autophagy-related cell death has the characteristics of LDH and HMGB1 release into the circulation. Necrosis was found in the liver, with no immune cell infiltrations, at 6 h post injection (Figure 5B). Therefore, the effect of IFN-γ on Con A-treated hepatocytes must be direct, and is not mediated by the enhancement of immune cells. Our in vitro study demonstrates that IFN-γ can enhance the Con A-stimulated autophagic flux to cause lysosomal cell death.

A massive breakdown of lysosomes could cause cell death by necrosis, which is related to increase of cytosolic acidification [10]. IFN-γ can regulate lysosome activities by the alteration of vacuolar pH or the activation of cathepsin expression to cause cell death [23], [24]. Here we found no significant increase in hepatocytes' acidification and protein level of cathepsin B/L upon IFN-γ stimulation. Instead, small increase in cell acidification can be detected at 6 hour post Con A treatment, which might lead to activate cathepsins in lysosome (Figure S4.) However, this increase in acidification is not enhanced by IFN-γ, suggesting that other factors are involved in IFN-γ/Con A-caused necrosis. In this study we further demonstrate that IFN-γ enhances the autophagic flux induced by Con A to cause LMP-associated necrotic cell death in hepatocytes. In addition, the interferon-induced GTPase, IRGM1, plays a crucial role to mediate this autophagy-associated necrosis. IRGM1 is reported to recruit to pathogen-containing phagosomes and autophagosomes, and to facilitate the acidification of theses vacuoles and their fusion with lysosomes [16], [25]. However, we did not observe IRGM1 in autophagosomes in IFN-γ/Con A-treated hepatocytes (data not shown). Instead, IFN-γ/Con A treatment enhances or sustains the IRGM1 expression and causes its redistribution to mitochondria and lysosomes, resulted in lysosome membrane disruption. The redistribution of IRGM1 to lysosomes has also been reported by Zhao et al. in IFN-γ-induced mouse embryonic fibroblasts [26]. How IRGM1 regulates lysosomal membrane permeabilization remains further investigation. Human IRGM1 recently been reported to regulate IFN-γ-induced autophagy by binding mitochondrial lipid to control mitochondrial fission [27]. We have reported that accumulation of Con A on mitochondria after endocytosis causes mitochondrial damage and autophagy of damaged mitochondria [18]. In this study, we further showed that co-localized of damaged mitochondria with IRGM1 in IFN-γ-treated murine hepatocytes, and its translocation to lysosome. Both Con A and IRGM1 binding to mitochondria might increase mitochondria membrane damage and enhance autophagolysosome process. Here we also found that Con A can up-regulate IRGM1 expression in hepatocytes (Figure 4A). Since BNIP3 is an important mediator to Con A-induced autophagy, the interaction of IRGM1 and BNIP3 in regulating autophagy process is worth to further study. The lysosome-mediated cell death induced by IFN-γ/Con A in hepatocytes causes HMGB1 release. HMGB1 can act as an inflammatory mediator to aggravate to inflammation-related disease [28]. The autophagy-regulated HMGB1 release in tumor cells, either in cell culture or in the serum of Con A-injected wild type mice, is consistent with the observation of Thurhurn J et al. [29].

Cell death with apoptotic or autophagic features can lead to different immune responses [30]. Clearance of apoptotic cells is considered to induce an anti-inflammatory reaction by production of transforming growth factor-β (TGF-β), prostaglandin E2, and platelet activating factor (PAF) [31]. Apoptotic cell death induced by most chemotherapeutic drugs and radiation in cancer therapy creates an immunosuppressive environment around the tumor site, which may promote further tumor growth. Cell death with autophagy stimulates a pro-inflammatory response that subsequently activates the immune system and facilitates efficient antigen cross-priming to CD8^+^ T cells [32], [33]. We have reported that Con A with both autophagic induction and immuno-modulation can generate a CD8^+^ T cell-dependent anti-hepatoma effect in mice [34]. These findings therefore support the use of induction of autophagy as a new target for tumor therapy. The release of HMGB1 post IFN-γ/Con A treatment by lysosomal cell death will provide a link to further anti-hepatoma CD8^+^ cytotoxic T cell activation. This is worthy of further investigation.

## Materials and Methods

### Reagents and antibodies

Con A, pepstatin A, 3-methyladenine (3-MA) and E64D were purchased from Sigma-Aldrich (St. Louis, MO, USA). The cell-permeable cathepsin L inhibitor, Z-Phe-Phe-CH_2_F, and cathepsin B inhibitor, CA-074Me, were purchased from Calbiochem (La Jolla, California, USA). Control, cathepsin B and IRGM1 siRNA were purchased from Sigma-Aldrich. Microtubule-associated proteins light chain 3 (LC3) and cathepsin L siRNA were purchased from Santa Cruz (Santa Cruz, CA, USA). The antisera used were: anti-signal transducer and activator of transcription 1 (STAT1) and phospho-STAT1 (Tyr 701) from Cell Signaling (Beverly, MA), anti- LC3 from MBL (Nagoya, Japan), anti-mitochondrial preprotein translocases of the outer membrane 20 (Tom20) and anti-green fluorescent protein (GFP) from Santa Cruz, anti-Bcl-2/adenovirus E1B 19 kDa-interacting protein 3 (BNIP3) from Sigma-Aldrich, anti-cathepsin B and anti-cathepsin L from R&D system (Minneapolis, MN, USA), anti-lysosomal-associated membrane protein 1 (LAMP 1) from ebioscience (San Diego, CA, USA), anti-immunity-related GTPase (IRGM), anti-high mobility group box 1 protein (HMGB1) and anti-β-actin from Abcam (Cambridge, UK). The recombinant human and murine interferon gamma were purchased from Peprotech (Rocky Hill, NJ, USA).

### Cell culture

The murine hepatoma cell line, ML-1_4a_ cell, was adapted from ML-1 cells in BALB/c mice as previously described [18]. HepG2 cells were obtained from the Cell Collection and Research Center (CCRC, Hsin-Chu, Taiwan). In inhibition experiments, the cells were pre-treated with several inhibitors, including pepstatin A (10 µg/ml), E64D (10 µg/ml), 3-MA (2 mM), Z-Vad-fmk (100 µM), bafilomycin A1 (2 nM), AG490 (25 µM), Z-Phe-Phe-CH_2_F (2 nM), and CA-074Me (50 µM), 1 h before addition of Con A (20 µg/ml) or IFN-γ (500 U/ml). Cell death was determined by propidium iodide exclusion staining and analyzed by flow cytometry. To detect the release of lactate dehydrogenase (LDH) from cells, the cell culture supernatants were collected for a LDH activity assay according to the guidelines of the detection assay kit (Roche Diagnostics, Mannheim, Germany). To monitor the status of lysosomal stability, ML-1_4a_ cells were stained with 1 µg/ml acridine orange at 37°C for 30 minutes. The red acridine orange fluorescence was visualized by a fluorescent microscope or analyzed by flow cytometry. ML-1_4a_ cells were planted on a cover-slide and transfected with pGFP-LC3 or siRNAs using Lipofectamine 2000 (Invitrogen Corp., Carlsbad, CA). Cells were fixed and stained with primary antibody against LC3, IRGM1, Tom20, LAMP-1, cathepsin B or cathepsin L followed by secondary antibody conjugated with Alexa Fluor 488 or 594. The cellular distribution of proteins was observed under a confocal fluorescence microscope (Olympus FV 1000, Japan).

### Mice

C57BL/6 mice were provided by the Animal Center of National Cheng Kung University (Tainan, Taiwan), and maintained in the pathogen-free facility of the Animal Laboratory of National Cheng Kung University. The IFN-γ^−/−^ mice on a C57BL/6 background were obtained from The Jackson Laboratory (Bar Harbor, ME). All animals were raised and cared for according to the guidelines set up by the National Science Council, ROC. The mouse experiments were approved by the institutional animal care and use committee of National Chen Kung University (IACUC approval No. 99159).To induce hepatitis, mice were injected intravenously with Con A at a dose of 40 mg/kg body weight, the serum being collected at various time points post injection. The serum alanine aminotransferase (ALT) was determined by using the Hitachi type 717 automatic analyzer (Hitachi, Tokyo).

### Western blot analysis

Cell lysates were prepared by extracting proteins with lysis buffer (Cell Signaling, Beverly, MA). Proteins were separated by SDS-PAGE and transferred to PVDF membranes. The membranes were blocked and incubated with primary antibodies. After incubation with peroxidase-conjugated secondary antibodies, the blots were visualized by enhancing chemiluminescence reagents (PerkinElimer Life Sciences, Boston, MA).

### Analysis of cytosolic cathepsin B/L activity

To measure the cytosolic cathepsin B/L activity, the cytosolic fraction was prepared as previously described [35]. In brief, the treated or non-treated cells were harvested and washed with phosphate buffered saline (PBS) twice. The digitonin-based extraction buffer (50 µg/ml digitonin, 250 mM sucrose, 20 mM HEPES, 10 mM KCl, 1.5 mM MgCl_2_, 1 mM EDTA, 1 mM EGTA, pH 7.5) was added to cells and put on ice for 30 minutes. The cytosolic fraction was then collected to measure cathepsin B/L activity by InnoZymeTM Cathepsin L Fluorogenic Activity Kit (Calbiochem) or to detect cathepsin B/L protein expression by Western blotting as described above.

## Supporting Information

Figure S1 **Electron micrographs of Con A-treated ML-1_4a_ cells**. ML-1_4a_ cells were pretreated with 3-methyladenin (3-MA) for 30 minutes and incubated with Con A for another 12 hours. A. The electron micrographs (×10000) of PBS-, Con A-, 3-MA- and Con A/3-MA-treated ML-1_4a_ cells are presented. B. The selected-areas of electron micrographs (×60000) from Con A-treated ML-1_4a_ cells are presented. M: mitochondria; AP: autophagosome.
(TIF)

Figure S2 **Analysis of apoptotic cells with sub G1 population in IFN-γ/Con A-treated ML-1_4a_ cells.** ML-1_4a_ cells were treated with PBS, Con A, IFN-γ, IFN-γ/Con A or puromycin for 24 hours. The cells were fixed with ethanol and stained with PI to analyze the sub G1 population by flow cytometry.
(TIF)

Figure S3 **Autophagy contributes to IFN-γ/Con A-induced necrotic cell death.** ML-1_4a_ cells were pretreated with DMSO or 3-methyladenin for 30 minutes and incubated with PBS, Con A, IFN-γ, or IFN-γ/Con A for another 24 hours. The cells were harvested and stained with Annexin V-FITC/PI. The cell population was analyzed by flow cytometry.
(TIF)

Figure S4 **Acidification and total cathepsin B/L protein expression in IFN-γ/Con A-treated hepatocytes.** **A**. ML-1_4a_ cells were pretreated with DMSO or bafilomycin A1 (Baf) and incubated with PBS, Con A, IFN-γ, or IFN-γ/Con A for another 6 hours. These cells were stained with AO for 30 minutes and analyzed the acidification by flow cytometry. **B**. ML-1_4a_ cells were with PBS, Con A, IFN-γ, or IFN-γ/Con A for 12 hours and extracted the total cell lysates. The expression of cathepsin B and L were determined by Western blot.
(TIF)
