# Supplementary figures and images for: Concanavalin A/IFN-Gamma Triggers Autophagy-Related Necrotic Hepatocyte Death through IRGM1-Mediated Lysosomal Membrane Disruption
Source: PLoS One. 2011 Dec 5;6(12):e28323. doi: 10.1371/journal.pone.0028323 (PMC3230628; doi:10.1371/journal.pone.0028323)

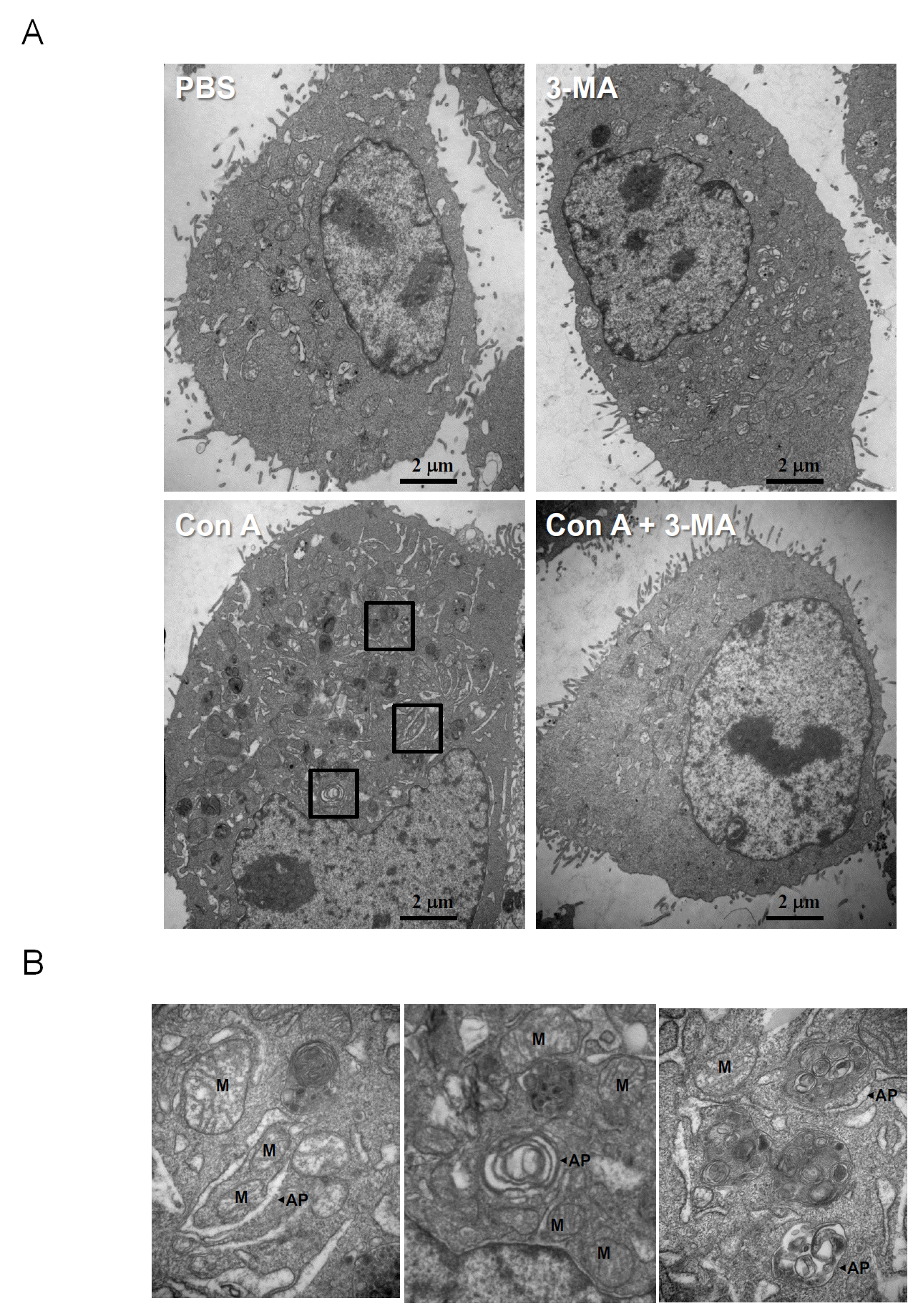

Supplement: Figure S1 — Electron micrographs of Con A-treated ML-14a cells. ML-14a cells were pretreated with 3-methyladenin (3-MA) for 30 minutes and incubated with Con A for another 12 hours. A. The electron micrographs (×10000) of PBS-, Con A-, 3-MA- and Con A/3-MA-treated ML-14a cells are presented. B. The selected-areas of electron micrographs (×60000) from Con A-treated ML-14a cells are presented. M: mitochondria; AP: autophagosome. (TIF) [file pone.0028323.s001.tif]

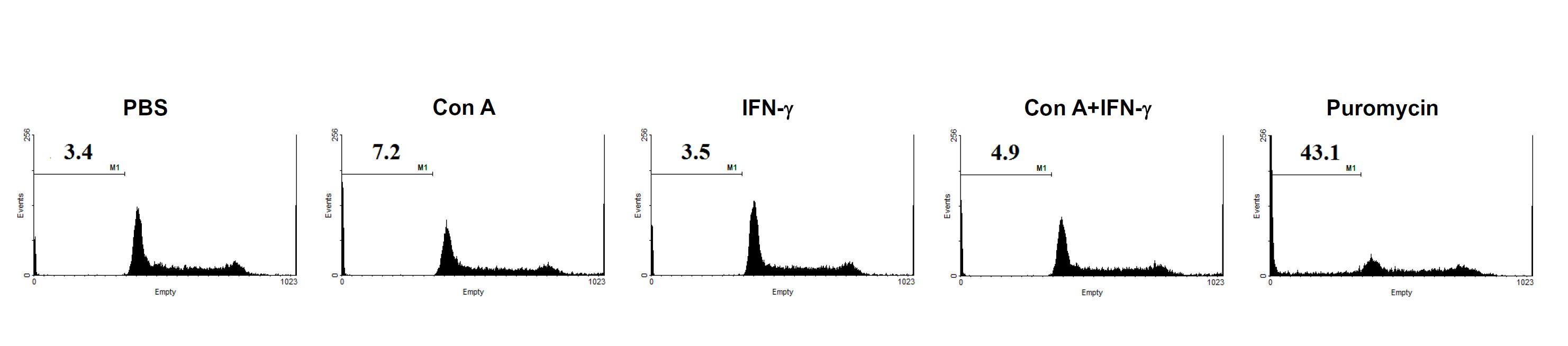

Supplement: Figure S2 — Analysis of apoptotic cells with sub G1 population in IFN-γ/Con A-treated ML-14a cells. ML-14a cells were treated with PBS, Con A, IFN-γ, IFN-γ/Con A or puromycin for 24 hours. The cells were fixed with ethanol and stained with PI to analyze the sub G1 population by flow cytometry. (TIF) [file pone.0028323.s002.tif]

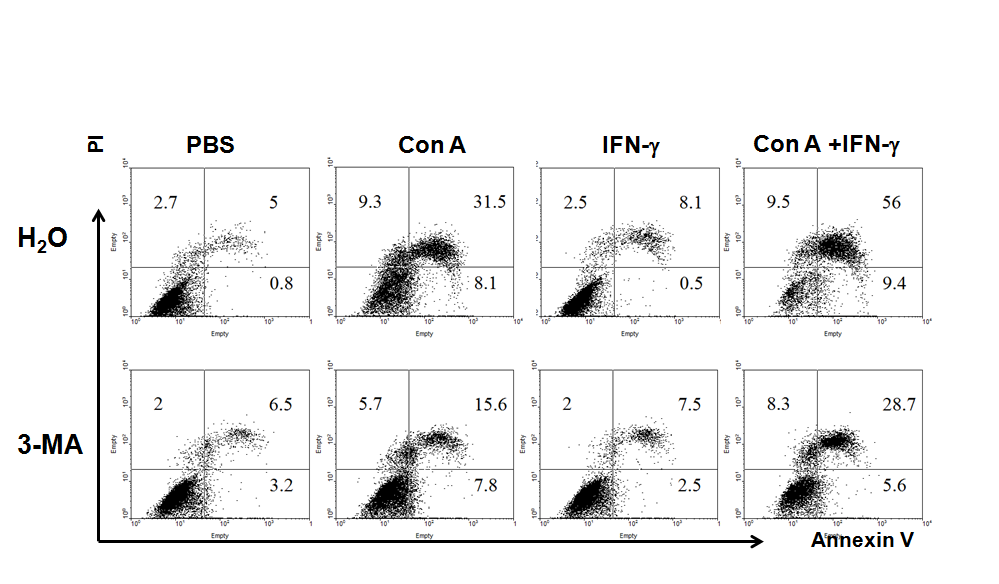

Supplement: Figure S3 — Autophagy contributes to IFN-γ/Con A-induced necrotic cell death. ML-14a cells were pretreated with DMSO or 3-methyladenin for 30 minutes and incubated with PBS, Con A, IFN-γ, or IFN-γ/Con A for another 24 hours. The cells were harvested and stained with Annexin V-FITC/PI. The cell population was analyzed by flow cytometry. (TIF) [file pone.0028323.s003.tif]

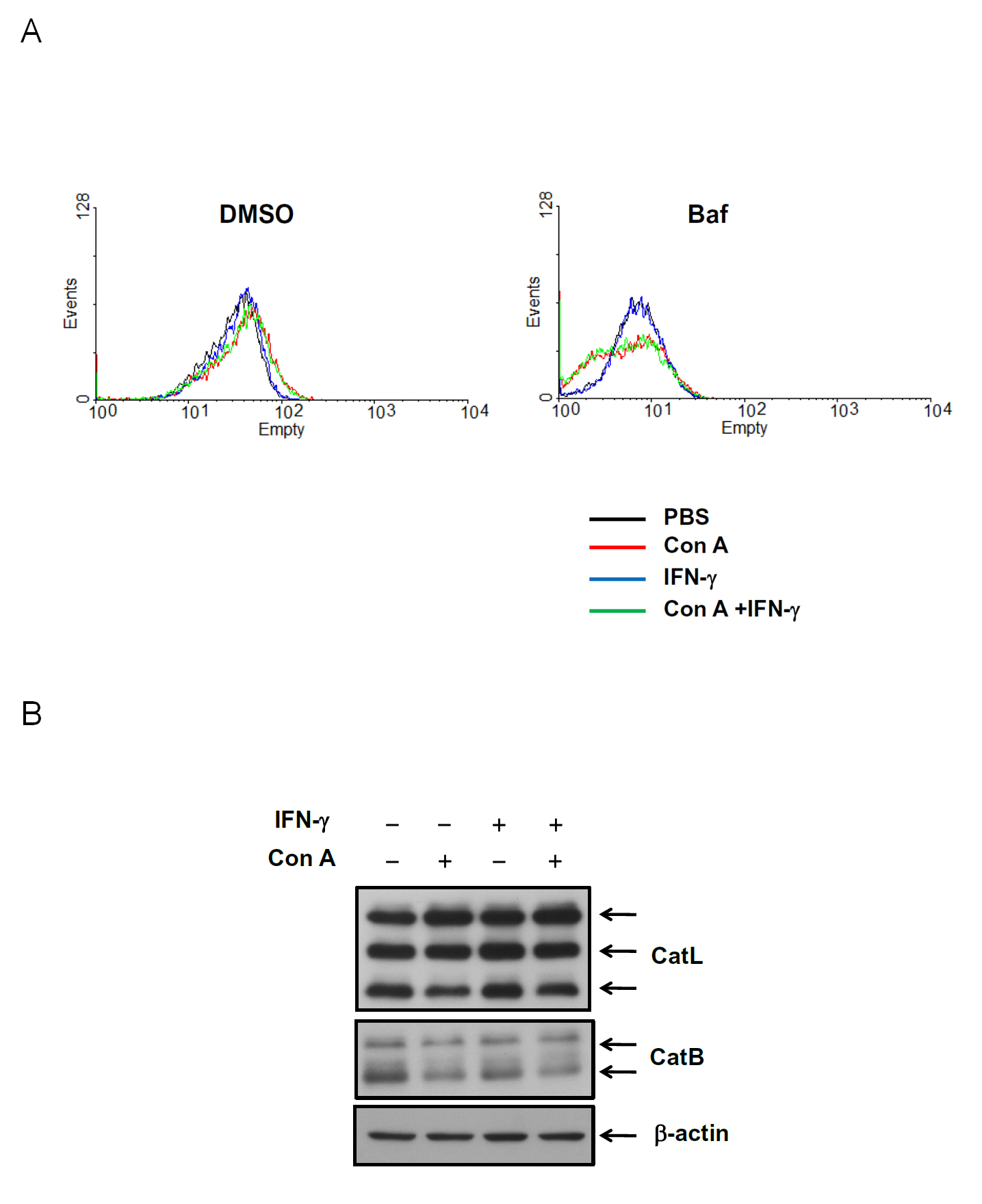

Supplement: Figure S4 — Acidification and total cathepsin B/L protein expression in IFN-γ/Con A-treated hepatocytes. A. ML-14a cells were pretreated with DMSO or bafilomycin A1 (Baf) and incubated with PBS, Con A, IFN-γ, or IFN-γ/Con A for another 6 hours. These cells were stained with AO for 30 minutes and analyzed the acidification by flow cytometry. B. ML-14a cells were with PBS, Con A, IFN-γ, or IFN-γ/Con A for 12 hours and extracted the total cell lysates. The expression of cathepsin B and L were determined by Western blot. (TIF) [file pone.0028323.s004.tif]
